# *Bifidobacterium animalis* subsp. *lactis* CECT 8145 BPL1^®^ Laxative Effects in Loperamide-Induced Constipated SD Rats

**DOI:** 10.3390/nu18081237

**Published:** 2026-04-14

**Authors:** Andrea Rodenes-Gavidia, Anna Mas-Capdevilla, Adrián Florit, María Enrique López, Daniel González-Hedström, Araceli Lamelas, Patricia Martorell, Empar Chenoll, Vanessa Illescas-Armijo, Juan Martínez-Blanch, Anna Antolín, Juan María Alcaide-Hidalgo, Roger Mariné-Casadó, Antonia Rojas, Laura Rago

**Affiliations:** 1 ADM R&D Health & Wellness, Parque Científico Universitat de València, Carrer del Catedràtic Agustín Escardino Benlloch, 9, 46980 Paterna, Spain; andrea.rodenes@adm.com (A.R.-G.); adrian.florit@adm.com (A.F.); maria.enrique@adm.com (M.E.L.); daniel.gonzalezhedstrom@adm.com (D.G.-H.); araceli.lamelas@adm.com (A.L.); patricia.martorell@adm.com (P.M.); maria.chenoll@adm.com (E.C.); vanessa.illescas@adm.com (V.I.-A.); juan.martinezblanch@adm.com (J.M.-B.); laura.rago@adm.com (L.R.); 2 PhD Programme in Biotechnology, Universitat Politècnica de València, Cami de Vera s/n, 46022 Valencia, Spain; 3 Eurecat, Centre Tecnològic de Catalunya, Technological Unit of Nutrition and Health, Avinguda Universitat 1, 43204 Reus, Spain; anna.mas@eurecat.org (A.M.-C.); anna.antolin@eurecat.org (A.A.); juanmaria.alcaide@eurecat.org (J.M.A.-H.); roger.marine@eurecat.org (R.M.-C.); 4 CIBER in Physiopathology of Obesity and Nutrition (CIBEROBN), Carlos III Health Institute, 28029 Madrid, Spain

**Keywords:** *Bifidobacterium animalis* subsp. *lactis* CECT 8145, constipation, probiotics, gut motility, microbiome modulation

## Abstract

**Background:** Constipation is a common gastrointestinal (GI) state for which probiotics have shown promise as a relief. This study examined the laxative effects of the strain *Bifidobacterium animalis* subsp. *lactis* CECT 8145 (BPL1^®^) in a loperamide-induced rat model of constipation. **Methods:** Fifty-nine rats were divided into control and loperamide-induced constipation groups. Animals received a 3-day intervention with either placebo or probiotic BPL1^®^ at two doses: 1.5 × 10^8^ CFU (colony-forming units) (low) and 3 × 10^9^ CFU (high). The study assessed several parameters to determine the probiotic’s effect, including: stool and gut characteristics, gastrointestinal transit time (GTT), gene expression and gut microbiome composition. **Results**: While loperamide significantly decreased stool number, weight and humidity, BPL1^®^ supplementation effectively restored these parameters, being more pronounced at a high dose. Microbiome analysis showed that BPL1^®^ at a low dose reduced the abundance of *Muribaculaceae* and *Muribaculum gordoncarteri*, associated with constipation. In addition, *Muribaculaceae* abundance was negatively correlated with stool humidity. Functional microbiome profiling indicated that BPL1^®^ suppressed pathways related to mucin degradation, vancomycin resistance and isoleucine biosynthesis while promoting L-lactate and pyridoxal-P (vitamin B6) biosynthesis, which may support gut motility and barrier integrity. **Conclusions:** *Bifidobacterium animalis* subsp. *lactis* BPL1^®^ exhibits potential as a functional probiotic for relieving constipation through improving stool excretion and consistency, inducing taxonomic changes and beneficial functional modulation of the intestinal microbiome. These findings justify further investigation into the mechanisms of BPL1^®^ as a probiotic for constipation management.

## 1. Introduction

Constipation, as defined by the Rome IV criteria, is characterized by the presence of two or more symptoms, such as straining, lumpy or hard stools, sensation of incomplete evacuation, anorectal blockage, manual maneuvers to facilitate defecation, or fewer than three spontaneous bowel movements per week, persisting for at least 12 weeks over the preceding 12 months [1]. Several biochemical, hormonal, and microbiological factors influence peristaltic movement, which makes constipation a complex multifactorial condition that is difficult to treat.

The global prevalence of constipation is estimated to be 14%, with a range of 2% to 27% in the United States and up to 80% in Europe, depending on the population studied and the definitions used [2,3,4,5]. Women, children, and the elderly are particularly affected, with lifestyle, diet, psychological, and social factors contributing to an increasing incidence of constipation in modern times [6]. Chronic constipation can significantly impair quality of life, highlighting the need for effective treatment strategies.

The pathophysiology of constipation involves alterations in colonic motility, neural regulation, and mucosal function [7,8]. The autonomic nervous system, particularly parasympathetic cholinergic signaling via muscarinic acetylcholine receptors (mAChRs), plays a key role in coordinating peristaltic contractions. The M2 and M3 receptor subtypes are predominant in gastrointestinal (GI) smooth muscle and mediate contraction through complex biochemical and electrical signaling cascades; reduced M3 receptor activity has been associated with decreased motility and constipation [9,10]. In parallel, aquaporins (AQPs), especially AQP3 and AQP8, regulate water transport in the colon and maintain stool hydration; their downregulation contributes to harder stools and delayed transit [11,12,13]. Mucin secretion by goblet cells also provides intestinal lubrication, and reduced mucin content has been reported in experimental models of constipation [11]. Consequently, stool parameters (weight, frequency, and water content), gastrointestinal transit time (GTT), colonic histology, mucin expression, and regulation of *AQP* and *mAChR* genes are key indicators for evaluating laxative efficacy.

Emerging evidence underscores the critical role of the gut microbiota in maintaining intestinal motility and bowel function. Constipation is often accompanied by dysbiosis, characterized by reduced levels of beneficial bacteria such as *Bifidobacterium* spp., *Lactobacillus* spp., *Prevotella* spp., and butyrate-producing genera and increased abundance of *Coprococcus* spp., *Ruminococcus* spp., *Akkermansia* spp., and *Clostridium* spp. [14,15]. Research using murine models of constipation also reported dysbiosis in gut microbiota with an increase in *Muribaculaceae* and a decrease in *Alistipes* and *Ruminococcus* [16]. These microbial alterations affect the production of short-chain fatty acids (SCFAs) which modulate colonic motility via cholinergic and serotonergic pathways [17,18,19]. Additionally, microbial metabolism of bile acids and interactions with enterochromaffin cells influence serotonin biosynthesis, further linking gut microbial composition to peristaltic regulation [20,21].

Some options for constipation management such as osmotic and stimulant laxatives, prokinetics, and secretagogues are often associated with adverse effects including abdominal discomfort and diarrhea, limiting long-term compliance [22]. Consequently, there is growing interest in probiotic-based interventions as safer, physiology-oriented alternatives capable of restoring microbial balance and improving intestinal motility.

Among probiotics, several *Bifidobacterium* and *Lactobacillus* strains have demonstrated efficacy in alleviating constipation symptoms through modulation of gut microbiota composition, enhancement of mucin secretion, and regulation of intestinal transit [23,24]. *Bifidobacterium animalis* subsp. *lactis* CECT 8145 (BPL1^®^) is a well-characterized probiotic strain with established safety and documented benefits in metabolic regulation, gut barrier integrity, and anti-inflammatory activity [25,26,27]. However, its potential role in constipation relief remains unexplored. To address this gap, the present pilot study investigates the effects of *B. animalis* subsp. *lactis* CECT 8145 (BPL1^®^) in a loperamide-induced rat model of constipation. The research integrates physiological, histological, molecular, and microbiological analyses—including stool characteristics, GTT, colonic structure, mucin secretion, expression of *AQP* and *mAChR* genes, and gut microbiota composition and function—to elucidate the mechanisms underlying the probiotic’s potential action in constipation attenuation.

## 2. Materials and Methods

### 2.1. Loperamide-Induced Constipation Model in Rats

#### 2.1.1. Experimental Design for the Animal Study

All procedures were approved by the Animal Ethics Committee of UTNS-REUS (Reus, Spain) and the Generalitat de Catalunya, following the Guide for the Care and Use of Laboratory Animals and the European Directive 86/609/EEC on the protection of animals used for experimental and scientific purposes.

The study involved 60 seven-week-old male Sprague–Dawley (SD) rats (Envigo, Barcelona, Spain), housed individually under temperature-controlled conditions (22 °C, 55% relative humidity) with a photoperiod of 12 h light/dark cycle. One rat died naturally (i.e., not as a result of any procedure performed during the study) halfway through the study and was therefore excluded from the total number of rats in the study (*n* = 59). Animals had ad libitum access to a standard diet (2014 Teklad Global 14% Protein Rodent Maintenance Diet; Envigo) and tap water. After a one-week adaptation period, the eight-week-old animals were randomly distributed into three experimental groups.

A sample size calculation was performed using G*Power v3.1.9.7 (two-tailed t test for independent means). The analysis was based on fecal pellet number, using mean and standard deviation values reported for the loperamide control and treated groups in Kim et al. [28]. With an effect size of 1.68, α = 0.05, and power = 0.90, the minimum required sample size was 9 animals per group. As 10 animals per group were included, the study can be considered adequately powered while remaining consistent with the principle of reduction. The additional animal per group provided a margin to account for potential animal loss or exclusion.

**Figure 1:**
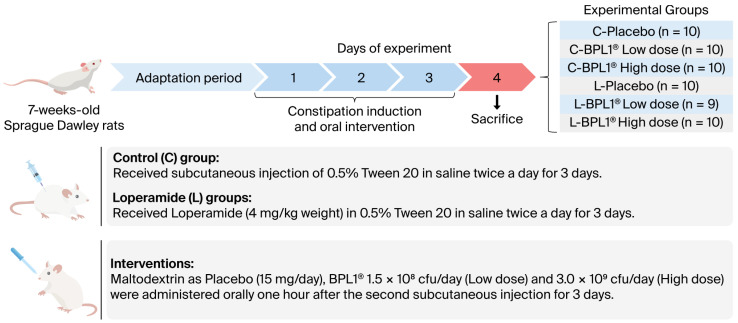
Experimental design and description of the experimental groups included in the present study. BPL1^®^: *B*. *animalis* subsp. *lactis* CECT 8145.

To carry out the constipation model, the experimental design described by Kim J.E. et al. [11,28], with minor modifications, was followed. Constipation was induced in rats (*n* = 29) by subcutaneous injection twice a day for 3 days of loperamide (4 mg/kg weight) in 0.5% Tween 20 in saline solution, whereas the control groups (*n* = 30) were injected with 0.5% Tween 20 in saline solution alone. The ingredient was administered after constipation had already been induced, as described by previous studies [29]. Thus, one hour after the second subcutaneous injection, control (C) and loperamide (L) rats were orally administrated maltodextrin as a placebo (Placebo) (15 mg/day) and *Bifidobacterium animalis* subsp. *lactis* CECT 8145 (BPL1^®^) at different doses: 1.5 × 10^8^ CFU/day (BPL1^®^ Low dose) and 3 × 10^9^ CFU/day (BPL1^®^ High dose). All interventions were diluted in water and administered by voluntary consumption. The experimental design and group description are shown in Figure 1. Body weight, as well as food and liquid intakes, was recorded daily during the experiment.

#### 2.1.2. Measurement of Stool Parameters

Stool parameters including consistency, water content, number, weight, frequency of depositions, and GTT were assessed during the study.

##### Consistency and Water Content

Stools were collected at intervals of 0, 2, 4, 6 and 8 h (starting at 9 a.m. and finishing at 5 p.m.) for the evaluation of stool consistency and water content on the 3rd day of the experiment. Fresh and dry weight of stools and water content were recorded for each interval in all groups. Stool dry weight was obtained after drying them in an oven at 70 °C for 24 h. Water content was calculated as the difference between the fresh and dried stool weights and expressed as a percentage using the formula: (water content (mg)/fresh feces (mg)) × 100. There were no samples of C-BPL1^®^ High dose at 4–6 h, L-Placebo 6–8 h, or L-BPL1^®^ Low dose at 2–4 h and 6–8 h because none of the rats in these groups defecated during those intervals.

##### Number, Weight and Frequency of Depositions

Stool number, weight, and frequency of depositions were recorded during a 22 h period from the third to the fourth day of the experiment (sacrifice day). Animals were placed in clean cages with free access to food and water from 5 p.m. on the third day to the time of sacrifice (3 p.m. of fourth day). Stools were collected during this period, and the number of depositions and fresh and dry weight (after being dried in an oven for 24 h at 70 °C) were recorded for each animal. The frequency of depositions was determined by quantifying the number of stools produced by each animal during the first 2 h and the subsequent 22 h.

##### Gastrointestinal Transit Time (GTT) Analysis

GTT was assessed using the carmine red dye test, a safe and non-invasive method that has been extensively used [30,31]. At 19 h, a food-grade carmine red solution (60 mg/mL) at a dose of 3 mL/kg body weight (bw) was administered via an intragastric tube, and the animals were individually housed. Starting at 8 h in the morning on the following day, the stool samples were collected at 1 h intervals, and the presence of color in the feces was determined at each interval. During this evaluation, animals had free access to water and feed. GTT is defined as the time in which the feces appear for the first time with a well-defined red color.

#### 2.1.3. Endpoint Analyses: Histopathology, Gene Expression, and Microbiota Analysis

On the fourth day of the experiment, rats were sacrificed under anesthesia using pentobarbital sodium (60 mg/kg bw; Merck, Barcelona, Spain). Intestinal tissues (small intestine, large intestine, and cecum) were collected, washed with saline, weighed, frozen in liquid nitrogen and stored at −80 °C for subsequent analysis. A portion of the transverse colon was fixed in 10% formalin for histopathological evaluation.

##### Histopathology

For histopathological analysis, transverse colons were fixed in 10% formalin for 24 h, dehydrated in successive ethanol concentrations (70%, 96% and 100% *v*/*v*), cleared with xylol/dimethyl benzene and infiltrated with paraffin by inversion at 52 °C (Citadel 2000, Thermo Fisher Scientific, Waltham, MA, USA). Samples were embedded in a paraffin block (HistoStar, Thermo Fisher Scientific, Waltham, MA, USA), sectioned into 2 μm thick slices (Microm HM 355S, Thermo Fisher Scientific, Waltham, MA, USA) and mounted on slides (JP Selecta Paraffin Bath, Thermo Fisher Scientific, Waltham, MA, USA). Sections were stained with hematoxylin and eosin (H&E) using an automated staining system (Varistain Gemini ES, Thermo Fisher Scientific, Waltham, MA, USA). Morphological features of these sections were observed using a Leica DM750 microscope (Leica Microsystems, Wetzlar, Germany), and images were captured with a Leica ICC50W camera (Leica Microsystems, Wetzlar, Germany) using Leica Application Suite v3.3.0 software. Histopathological assessments included quantification of goblet cells (percentage of the total cells), evaluation of gut-associated lymphoid tissue (GALT) categorized as absent, mild, moderate, or severe, and germinal centers. Additional macroscopical qualitative analyses were also evaluated, such as identification of melanosis coli (presence of black or brown pigment in the colorectal mucosa associated with laxative use).

##### Gene Expression

For gene expression analysis, the total RNA was extracted from colon samples using Tripure Reagent (Qiagen, Barcelona, Spain) according to the manufacturer’s protocol. RNA yield was quantified on a NanoDrop 1000 spectrophotometer (Thermo Fisher Scientific, Waltham, MA, USA). Complementary DNA (cDNA) synthesis was performed using the High-Capacity cDNA Reverse Transcription Kit (Applied Biosystems, Barcelona, Spain) with a MyGene L Series Peltier Thermal Cycler (LongGene, Hangzhou, China). Quantitative reverse transcriptase polymerase chain reaction amplification was performed using LightCycler 480 SYBR green I Master-20 (Roche Diagnostics, Barcelona, Spain) in a Light Cycler 480II (Roche Diagnostics, Barcelona, Spain). The primers used for the different genes were obtained from Biomers.net (Biomers, Ulm, Germany) and are described in Table 1. Relative mRNA levels of *mAChRM2* and *mAChRM3*, *AQP3* and *AQP8*, and the housekeeping gene peptidylprolyl isomerase A (*PPIA*) were quantified by real-time PCR.

##### Microbiota Analysis

For fecal microbiota analysis, DNA was extracted from fecal samples using the QIAsymphony PowerFecal Pro DNA Kit (Qiagen, Barcelona, Spain) with a robotic magnetic bead-based kit. DNA concentration and quality were assayed using the NanoDrop Spectrophotometer (Thermo Fisher Scientific, Waltham, MA, USA) and the Qubit dsDNA System (Thermo Fisher Scientific, Waltham, MA, USA). DNA samples were normalized to generate the sequencing libraries with the Illumina DNA Library Prep kit (Illumina, San Diego, CA, USA) following the manufacturer’s instructions. Library quality control was ensured by profiling and length distribution analysis using the HSD5000 kit in the TapeStation 4200 equipment (Agilent Technologies, Santa Clara, CA, USA). The libraries were loaded in the NovaSeq 6000 sequencing platform (Illumina, San Diego, CA, USA) using 150 paired end reads attaining a minimum of 20 million reads per sample. The sequencer generated BCL files as the primary sequencing output (NovaSeq Control Software (NCS) v1.6). The Bcl2fastq v2.20 program (bcl2fastq (RRID:SCR_015058)) was used to convert the BCL (Binary Base Call) sequencing reads to FASTQ format, remove the sequencing adapter, and separate the sequences according to samples.

Bioinformatic analysis was performed to process sequencing data. Optical duplicates were removed using the Clumpify (BBMap v36.92) tool [32] and reads with a Phred quality score below 20 (Q20) and length shorter than 50 nucleotides (L50) were filtered out using the Reformat (BBMap v36.92) tool [32]. The host genome was filtered with the NGLess (v1.0.0-Linx64) [33] tool, using the reference genome of *Rattus norvegicus* mRatBN7.2 (GeneBank assembly GCA_015227675.2). Reads with alignments of more than 45 bases and 97% similarity to the reference genome were discarded. To ensure comparability between samples with more than 20 million sequences, they were randomly trimmed to this number with Seqtk v1.4-r122 [34]. Taxonomic profiling was performed using the Metaphlan v4.0 [35] pipeline and CHOCOPhlAn SGB vJun23 (mpa_vJun23_CHOCOPhlAnSGB_202307). The number of reads attributed to each clade was computationally calculated for all identified taxa.

Assembly of sequences was performed using the Megahit v1.2.9 assembler [36], with k-mer sizes of 21, 33, 55, 77, 99, and 127. The assembly obtained with k-mer 127 was selected for further analysis due to its higher number of contigs and longer sequence lengths. Contigs larger than 500 bp were used to predict the prokaryotic Open Reading Frames (ORFs) with Prodigal v2.6.3 [37]. ORFs without start and stop codons were discarded with Seqtk v1.4-r122 [34]. The final ORFs were quantified for each sample with Salmon v1.10.3 software [38]. Functional annotation was performed with the web server GhostKoala v2.0 [39] to obtain KEGG annotation, the dbCAN3 v3.0.7 tool [40] was used to annotate the CAZy (Carbohydrate active enzymes), and the BLASTp v2.15.0+ algorithm [41] and the internal BrainDB database were used to identify the genes associated with pathways related with constipation based on the literature.

### 2.2. Statistical Analysis

#### 2.2.1. Clinical Parameters

Statistical analyses of clinical parameters were performed using the IBM SPSS Statistics v25.0 program [42]. Grubbs’ test was used to detect outliers, which were excluded from subsequent analyses. Differences among all groups were evaluated using two-way ANOVA (2wANOVA). Significant ANOVA results were followed by Student’s *t*-test pairwise comparisons with *p*-values adjusted by the Benjamini and Hochberg method to control the false discovery rate [43]. The results are presented as the mean ± standard error of the mean (SEM). Statistical significance level was set at bilateral 5% (*p* < 0.05), while *p* < 0.1 was considered indicative of a trend toward significance.

#### 2.2.2. Metagenomic Analysis

Metagenomic data analysis was performed using RStudio v2024.04.2+764 software [44] and the programming language R v4.3.3 [45]. The relative taxonomic abundances of the samples were displayed with collapsed histograms plotted by the ‘ggplot2′ v3.5.1 library in R [46]. Taxa and gene data were normalized using the rarefaction technique from the ‘phyloseq’ v1.46.0 R package [47] for alpha diversity analysis. The richness, Shannon and Simpson indexes were calculated using the ‘vegan’ v2.6-6.1 R package [48]. Violin plots and boxplots were created with the ‘ggpubr’ v0.4.0 library in R [49] and a Wilcoxon signed-rank test was performed through the ‘stats’ v3.6.0 R package [50] to determine significance between groups.

Taxonomic dissimilarities based on species were illustrated using Principal Coordinates Analysis (PCoA) across the samples carried out using the Bray–Curtis distance matrix calculated with the ‘phyloseq’ v1.46.0 R package [47] and represented with the ‘ggplot’ v3.4.0 R package [46]. The effects of the factors on taxonomy data were evaluated with the PERMANOVA test with the ‘vegan’ v2.6-6.1 R package [48] using the Bray–Curtis dissimilarity matrix that was calculated from the relative abundances of functional categories across all samples.

Differential taxa and genes abundance analysis was conducted using the ‘DESeq2′ v1.42.0 R package [51]. Normalization was performed using the ‘Relative Log Expression’ method. The ‘EstimateSizeFactors’ function was used to calculate the scaling factors using the median ratio between taxa and gene abundances and the geometric mean. The ‘poscounts’ method was used to address the taxa and genes that had multiple zeros in the samples. A taxon was considered differentially abundant with a *p*-value adjusted by the Benjamini and Hochberg method [43] of less than 0.05—less than 0.1 was considered a tendency—and if it was present in at least 50% of the samples of one of the compared groups.

The MaAsLin2 v1.18.0 [52] tool was used to identify significant correlations between clinical variables and taxa abundance. This tool normalizes and transforms data, in order to establish multivariate associations between clinical and taxonomic data. To minimize the false positive rate, a *p*-value adjustment was performed using the FDR (false discovery rate) method [43]. Results with an adjusted *p*-value lower than 0.05 were considered significant.

Functional profile significance between groups was assessed using Gene Set Enrichment Analysis (GSEA) on the metagenomic dataset using the ‘fgsea’ v1.16 R package [53] on the KEGG module. Genes identified as statistically significant in the differential abundance analysis by DESeq2 were ranked using the ‘stat’ statistic for GSEA. The results were represented graphically by Heatmaps, which were built using the ‘ComplexHeatmap’ v2.11.1 R package [54].

## 3. Results

### 3.1. In Vivo Evaluation of Potential Laxative Effects of Bifidobacterium animalis *subsp.* lactis BPL1^®^ in Loperamide-Induced Constipated Rats

#### 3.1.1. Effect of BPL1^®^ on Feeding Behavior and Body Weight

To investigate whether BPL1^®^ administration could affect body weight and feeding behavior, we measured body weight gain and cumulative intake (food and liquid) in control (C) and loperamide-induced constipated (L) rats at the end of the study. All of these parameters remained consistent across all groups (Table 2).

#### 3.1.2. Effect of BPL1^®^ on Stool Parameters

Regarding stool consistency, all groups showed normal feces in all the measured intervals in the 8 h study and no differences were found in water content among the groups in the 8 h register (Table 3).

The effects of the interventions in control (C) and loperamide (L) rats regarding GTT were also evaluated. Although the GTT differed across the study groups (2wANOVA, *p* = 0.048), no significant differences were observed in the pairwise comparisons (Table 4).

**Figure 2:**
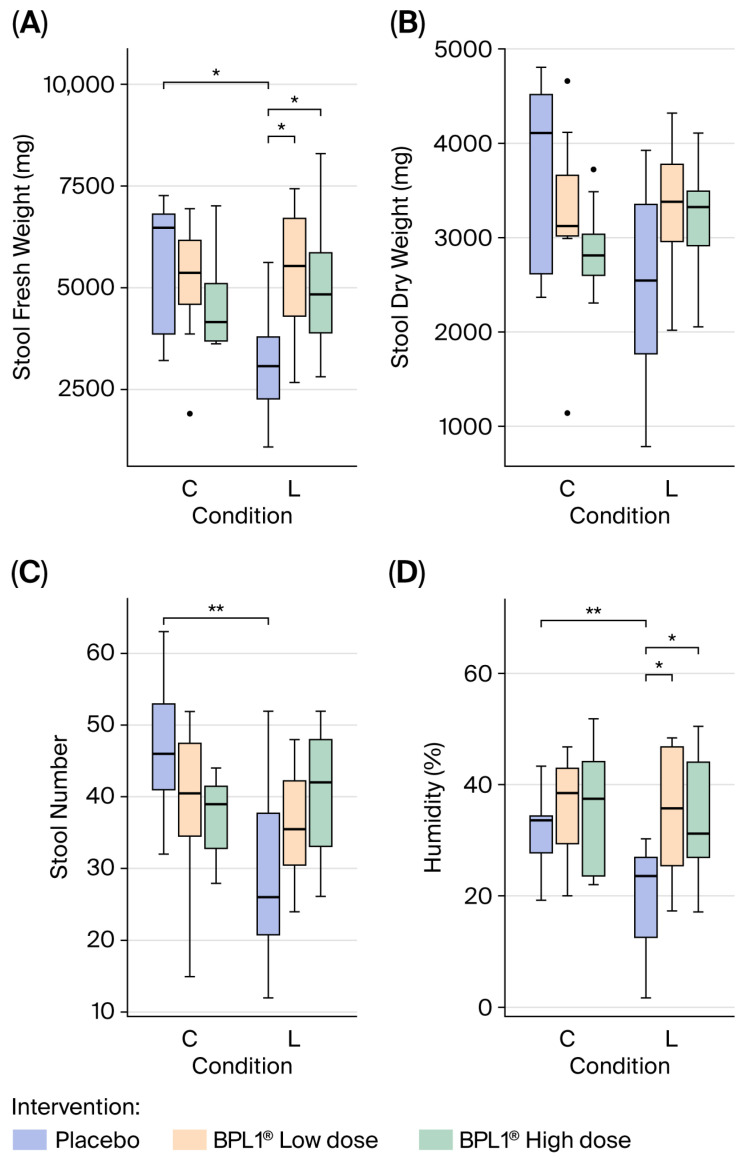
Stool weight (fresh and dry) (**A**,**B**), stool number (**C**) and humidity (**D**) in control (C) and loperamide (L) groups administered with the placebo or BPL1^®^ in low and high doses at 22 h register. Data distribution is represented by a boxplot. (*): *t*-test, adj. *p* < 0.05. (**): *t*-test, adj. *p* < 0.01.

Finally, stool number, weight (fresh and dry) and frequency were evaluated in a 22 h register from third to fourth day of the study. The loperamide-induced constipation model resulted in a significant change in the number of stools (2wANOVA, *p* = 0.0068), the fresh stool weight (2wANOVA, *p* = 0.0143) and humidity (2wANOVA, *p* = 0.0062), whereas the dry stool weight showed no significant changes (2wANOVA, *p* = 0.0717). In fact, significant differences were found between C and L groups administered with the placebo, reducing the stool number in the constipated group (*t*-test, adj. *p* = 0.0078), fresh stool weight (*t*-test, adj. *p* = 0.0102), and humidity (*t*-test, adj. *p* = 0.0208). In addition, when comparing loperamide groups, it was observed that in both doses of the BPL1^®^ probiotic, the loperamide effect in constipated animals was reverted. In this line, significant differences were observed between L-Placebo and L-BPL1^®^ Low dose for stool fresh weight (*t*-test, adj. *p* = 0.036) and humidity (*t*-test, adj. *p* = 0.03). The administration of BPL1^®^ at a high dose to loperamide-administered rats showed a laxative effect in constipated animals, being able to significantly increase fresh stool weight (*t*-test, adj. *p* = 0.048) and humidity (*t*-test, adj. *p* = 0.042) when compared to L-Placebo (Figure 2).

**Figure 3:**
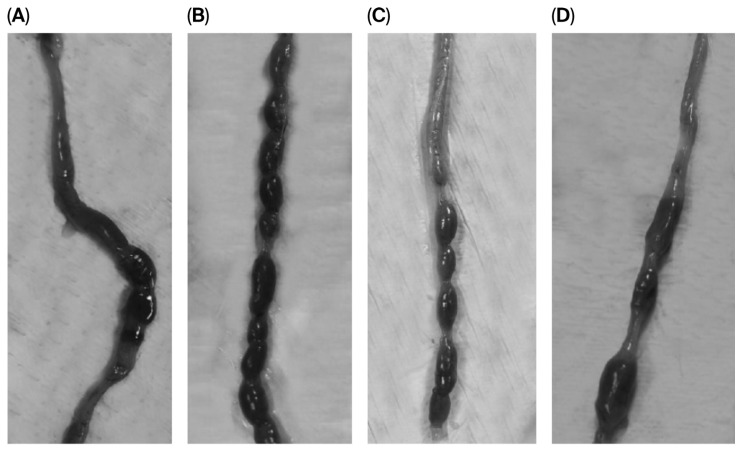
Macroscopical evaluation of transverse colon at the endpoint from the control (C) and the loperamide (L) groups receiving a placebo or low and high doses of BPL1^®^: (**A**) C-Placebo, (**B**) L-Placebo, (**C**) L-BPL1^®^ High dose and (**D**) L-BPL1^®^ Low dose.

Macroscopical qualitative analysis at the endpoint also revealed differences between the C-Placebo and L-Placebo groups, and among loperamide groups administered with the placebo or BPL1^®^ (low and high doses). It was observed that animals from the C-Placebo group presented a reduced number of stools compared to the L-Placebo group. Also, C-Placebo presented feces larger and more hydrated than those presented by L-Placebo. In turn, L-Placebo feces were smaller and more compact, indicating dehydration probably caused by the demonstrated prolonged transit time through colon. On the other hand, BPL1^®^’s laxative effects in L groups were also observed in the appearance of the animal colons. L-BPL1™ groups showed a lower number of feces than in the L-Placebo group, whereas feces from BPL1^®^-administered rats at both doses showed a similar appearance to the C-Placebo group (Figure 3).

#### 3.1.3. Effect of BPL1^®^ on Ultrastructure of the Transverse Colon

To investigate whether BPL1^®^ intervention could impact on the histological structure of the transverse colon, histological parameters were measured in H&E-stained transverse colons of control and loperamide rats (Table 5). Overall, there were no significant changes in goblet cell number (2wANOVA, *p* = 0.14), gut-associated lymphoid tissue (GALT) (2wANOVA, *p* = 0.90), or germinal centers (2wANOVA, *p* = 0.91) among the groups (Table 5).

### 3.2. Association Between the Laxative Effects of BPL1^®^ and Downstream Signaling Pathway of mAChRMs and AQPs

**Figure 4:**
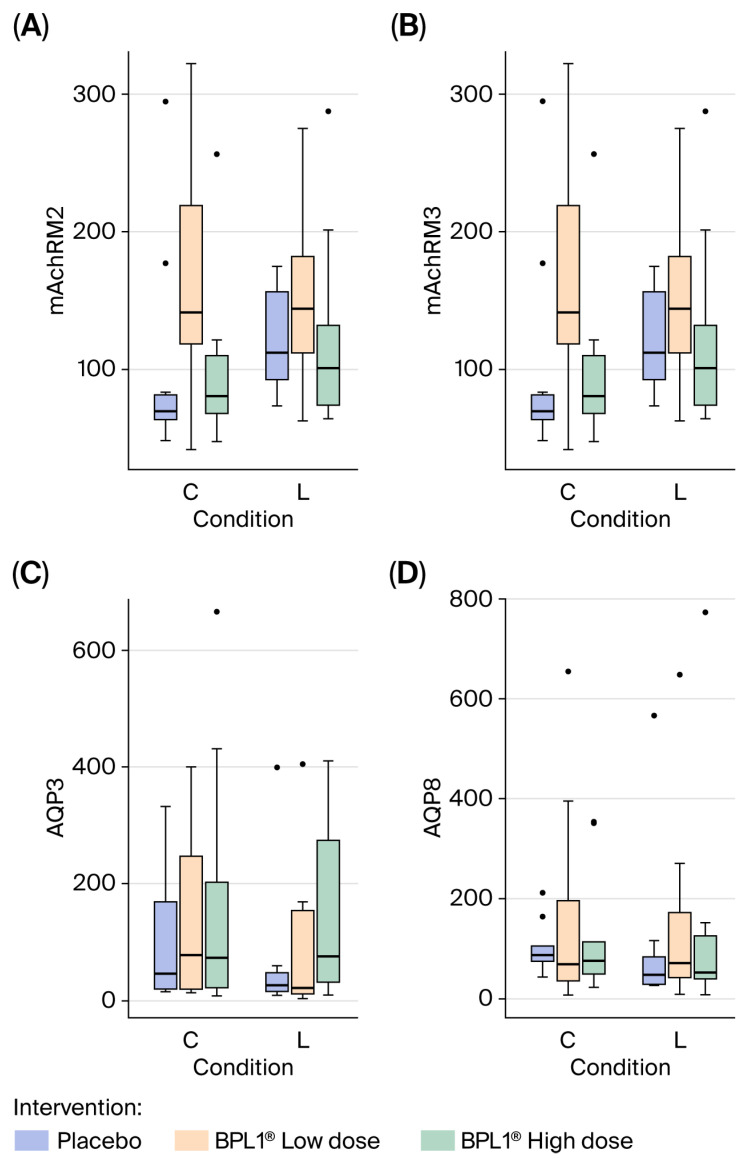
The mRNA expression levels of genes related to constipation in colon in the control (C) and the loperamide (L) groups: (**A**) *mAchRM2*, (**B**) *mAchRM3*, (**C**) *AQP3* and (**D**) *AQP8*. Data distribution is represented by a boxplot.

To further characterize the effects of BPL1^®^ on constipation, the expression levels of key constipation-associated genes were quantified, including *mAChRM2* and *mAChRM3*, as well as *AQP3* and *AQP8*. Overall, the ANOVA analysis revealed no significant differences across the studied groups for any of the four genes (*p* > 0.05) (Figure 4).

### 3.3. Gut Microbiota Modulation of BPL1^®^ on Constipated Murine Model

Shotgun metagenomic sequencing was performed to investigate the gut microbiome modulation in the constipated murine model during the intervention with the BPL1^®^ probiotic. A total of 268 different genera and 359 species were detected with the taxonomical classification of sequences. The bacterial community of this study was dominated by the genera *Bifidobacterium* (13.75 ± 13.12%), *Limosilactobacillus* (12.11 ± 7.46%), and *Lactobacillus* (11.55 ± 6.66%). Consistently, *Bifidobacterium pseudolongum* (13.74 ± 13.11%), *Limosilactobacillus reuteri* (12.11 ± 7.46%) and *Lactobacillus johnsonii* (11.52 ± 6.66%) were found as the most prevalent species (Figure S1).

Alpha diversity analysis revealed significant differences between studied groups for both richness and Shannon measures (2wANOVA, *p* = 0.0365 and *p* = 0.0347, respectively). In contrast, the Simpson index showed no significant differences (2w ANOVA, *p* = 0.0834). Furthermore, pairwise comparisons revealed no significant differences between the control and loperamide placebo groups. However, an increase in microbial diversity was observed in the control subjects that received the BPL1^®^ Low dose compared to placebo in both richness (*t*-test, *p* = 0.005) and Shannon index (*t*-test, *p* = 0.006) (Figure S2A). Additionally, within the BPL1^®^ Low dose cohort, the control group showed higher levels of richness and Shannon index compared to the constipated group (*t*-test: richness, *p* = 0.22; Shannon, *p* = 0.009). Furthermore, in the control groups, the BPL1^®^ High dose showed a significant increment in bacterial richness compared to the placebo group (*t*-test, *p* = 0.017).

To further understand the taxonomic dissimilarities between groups, we analyzed beta diversity based on Bray–Curtis distances. The PCoA plot revealed a dispersed distribution of samples across groups (Figure S2B). PERMANOVA analysis revealed significant differences in bacterial composition based on the condition (control and loperamide) (R^2^ = 0.07, *p* = 0.001). A tendency was also detected for the interaction between the condition and intervention variables (R^2^ = 0.045, *p* = 0.087). However, the intervention alone did not have a significant effect (R^2^ = 0.03, *p* = 0.493).

The subsequent step involved evaluating the taxa distribution and relative abundances at the genus and species level. At the genus level, a significant shift in gut microbiota composition was observed (Figure S4). In the placebo groups, the abundance of *Muribaculum* (Wald test, adj. *p* = 0.0364, log2FC = 2.41), *Lachnospiraceae* GGB28852 (Wald test, adj. *p* = 3.35 × 10^−15^, log2FC = 27.26), Bacteroidales unclassified (Wald test; GGB24132: adj. *p* = 0.0071, log2FC = 1.47; GGB27872: adj. *p* = 0.0374, log2FC = 1.94), and Bacteroidetes GGB14001 (Wald test, adj. *p* = 6.73 × 10^−5^, log2FC = 3.79) increased significantly in the loperamide group compared to the control, while unclassified *Lachnospiraceae* showed a significant decrease (Wald test: adj. *p* = 0.0119, log2FC = −2.07). Administration of BPL1^®^, at both low and high doses, reversed some of these changes in the loperamide-intervention group. Specifically, Bacteroidales (GGB24132 and GGB27872) and Bacteroidetes (GGB14001) (Wald test; GGB24132: adj. *p* = 0.0479, log2FC = −1.19; GGB27872: adj. *p* = 0.0334, log2FC = −2.09; GGB14001: adj. *p* = 0.0014, log2FC = −3.21) decreased their levels at low dose compared to placebo, while the high dose led to a marked reduction in *Lachnospiraceae* GGB28852 (Wald test; GGB28852: adj. *p* = 4.36 × 10^−20^, log2FC = −32.16).

In the control group, fewer changes were observed following probiotic administration. The BPL1^®^ Low dose group showed an increased abundance of Bacteroidetes GGB30302 compared to the placebo (Wald test, adj. *p* = 3.19 × 10^−6^, log2FC = 23.31), while the BPL1^®^ High dose group resulted in decreased abundances of *Limosilactobacillus* (Wald test, adj. *p* = 0.0121, log2FC = −2.08) and *Lactobacillus* (Wald test, adj. *p* = 0.0292, log2FC = −2.07) among others (Figure S4).

**Figure 5:**
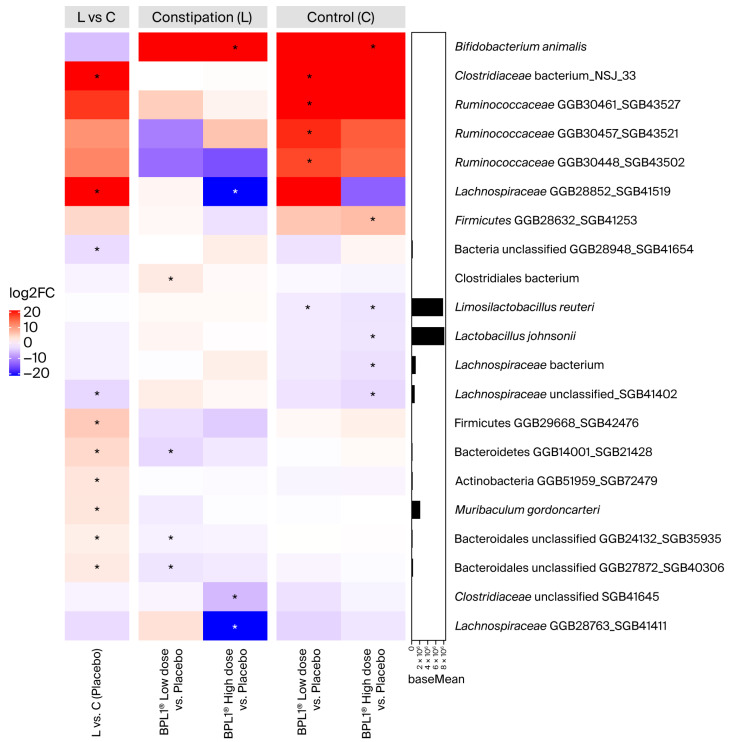
Differential abundance analysis of the microbial composition of murine fecal samples. Complex heatmap with the species differentially abundant according to the log2FC of the groups compared. Red color indicates that the species is over-represented in the first group of the comparison, while blue color indicates it is over-represented in the second group of the comparison. The barplot shows the mean normalized abundance (baseMean) of each taxon. Statistical significance was tested with “the DESeq2” package using its internal Wald test. (*): adj *p*-value < 0.05. In *, the presence of the taxon was found in at least 50% of samples of at least one of the compared groups. logFC, log fold change.

At the species level (Figure 5), findings were consistent with genus-level observations. In the loperamide placebo groups, significant increases were observed in *Muribaculum gordoncarteri* (Wald test, adj. *p* = 0.0335, log2FC = 2.56), *Clostridiaceae* NSJ_33 (Wald test, adj. *p* = 4.71 × 10^−44^, log2FC = 26.88), and two Bacteroidales unclassified (Wald test; GGB24132_SGB35935: adj. *p* = 0.0042, log2FC = 1.59; GGB27872_SGB40306: adj. *p* = 0.0218, log2FC = 2.07), with an unclassified *Lachnospiraceae* SGB41402 decrease (Wald test, adj. *p* = 0.0060, log2FC = −3.1968). Administration of BPL1^®^ in the loperamide animals reversed some of these changes. BPL1^®^ Low dose reduced the abundance of Bacteroidales unclassified species compared to L-Placebo (Wald test; GGB24132_SGB35935: adj. *p* = 0.0457, log2FC = −1.23; GGB27872_SGB40306: adj. *p* = 0.0229, log2FC = −2.10), while BPL1^®^ High dose reduced the abundance of *Lachnospiraceae* (Wald test; GGB28852_SGB41519: adj. *p* = 7.08 × 10^−18^).

The species-level changes differed between the control and loperamide-induced groups. In the control cohort, BPL1^®^ Low dose increased several *Ruminococcaceae* (Wald test; GGB30461_SGB43527: adj. *p* = 7.58 × 10^−7^, log2FC = 23.60; GGB30457_SGB43521: adj. *p* = 7.33 × 10^−5^, log2FC = 18.96; GGB30448_SGB43502: adj. *p* = 9.47 × 10^−5^, log2FC = 17.32) and *Clostridiaceae* species (Wald test, adj. *p* = 8.28 × 10^−31^, log2FC = 22.57), while *Limosilactobacillus reuteri* decreased (Wald test, adj. *p* = 0.0352, log2FC = −1.71). In contrast, BPL1^®^ High dose reduced the abundance of four species, including *Limosilactobacillus reuteri* (Wald test, adj. *p* = 0.0034, log2FC = −2.19) and *Lactobacillus johnsonii* (Wald test, adj. *p* = 0.0188, log2FC = −2.15).

Notably, BPL1^®^ High dose administration resulted in a significant increase in *Bifidobacterium animalis* compared to placebo in both control and loperamide groups (Wald test; adj. *p* = 1.80 × 10^−31^, log2FC = 29.74; adj. *p* = 1.94 × 10^−46^, log2FC = 37.09).

Due to the significant increase in *Muribaculum* and *M. gordoncarteri* abundance, a differential analysis of *Muribaculaceae* abundance in BPL1^®^ in induced loperamide rats was carried out. Significantly, the introduction of the low dose of the BPL1^®^ probiotic reduced the abundance of *Muribaculaceae* in the constipated animals compared to the L-Placebo group (Figure S3a). In addition, the abundance of this family decreased significantly with the percentage of fecal humidity (F test, adj. *p* = 0.0173, coef = −0.6036) (Figure S3b).

### 3.4. Impact of BPL1^®^ on Gut Microbiota Functionality

**Figure 6:**
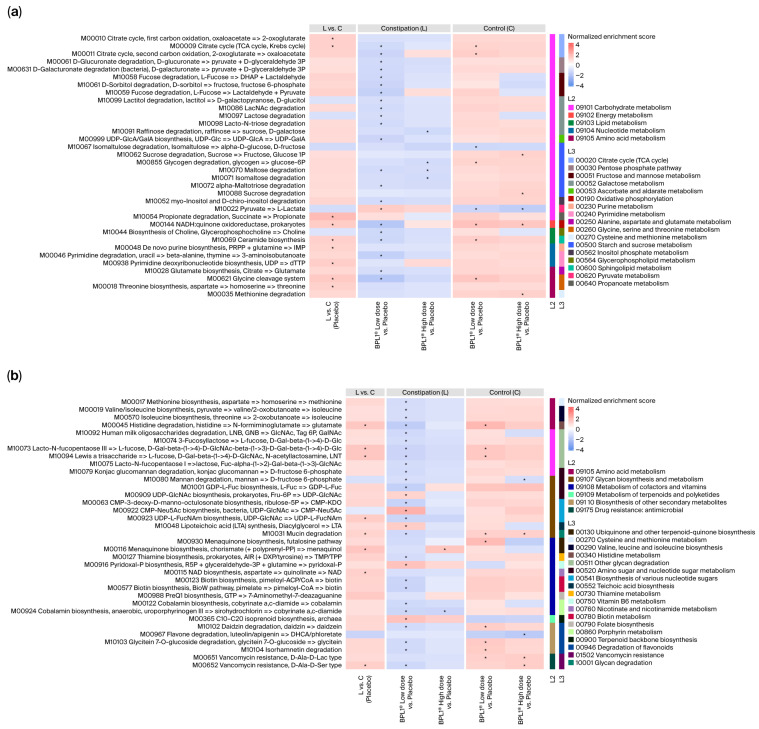
Heatmaps with the Normalized Enrichment Score (NES) of KEGG module abundances (**a**,**b**). Red color indicates positive NES values (NES > 0), which means the module is enriched in the first group of the comparison. Blue color indicates negative NES values (NES < 0), which means the module is enriched in the second group of the comparison. Legend L2 refers to KEGG annotation at the general category level, while L3 refers to KEGG annotation for specific pathway categories. (*): adj. *p*-value < 0.05. NES < −1.18 & >1.18.

Functional analysis was conducted to investigate the mechanisms underlying the effects of the BPL1^®^ probiotic intervention in the constipated model, focusing on the enrichment of KEGG functional modules using the GSEA method. In the placebo comparison, the constipated cohort exhibited significant enrichment in modules related to energy and lipid metabolism (e.g., M00144, adj. *p* = 0.0230, NES = 1.40; M10069, adj. *p* = 0.0163, NES = 1.25), mucin degradation (M10031, adj. *p* = 0.0317, NES = 1.18), cofactor and vitamin metabolism (e.g., M00116, adj. *p* = 0.0093, NES = 1.55), and vancomycin resistance (M00652, adj. *p* = 0.0439, NES = 1.30), among others (Figure 6). Administration of BPL1^®^ reversed these changes in the constipated cohort, with the low dose showing a more pronounced and statistically significant effect.

In the loperamide-induced groups, BPL1^®^ administration induced notable shifts in metabolic profiles, particularly with the low dose. Key enriched biosynthesis modules included L-lactate (M10022, adj. *p* = 0.0160, NES = 1.40), CMP-Neu5Ac (M00922, adj. *p* = 0.0019, NES = 2.08), Pyridoxal-P (M00916, adj. *p* = 0.0072, NES = 1.65), and isoprenoid biosynthesis (M00365, adj. *p* = 0.0335, NES = 1.65). Conversely, modules associated with methionine biosynthesis (M00017, adj. *p* = 0.0083, NES = −1.22), isoleucine biosynthesis (M00019, adj. *p* = 0.0314, NES = −1.19), CMP-KDO biosynthesis (M00063, adj. *p* = 0.0055, NES = −1.57), mucin degradation (M10031, adj. *p* = 1.97 × 10^−6^, NES = −1.33), and vancomycin resistance (M00652, adj. *p* = 0.0031, NES = −1.38) decreased significantly (Figure 6). The high dose of BPL1^®^ also reduced five functional modules involved in cobalamin biosynthesis (e.g., M00924, adj. *p* = 0.0023, NES = −1.48) and the degradation of raffinose, maltose, isomaltose, and alpha-maltotriose (e.g., M10091, adj. *p* = 0.0180, NES = −1.34). Meanwhile, menaquinone biosynthesis was enriched (M00116, adj. *p* = 0.0187, NES = 1.54) (Figure 6).

In the control cohort, BPL1^®^ administration (both low and high doses) produced fewer changes compared to placebo, showing a similar pattern to the placebo comparison. Significant enrichment was observed in modules related to energy metabolism (e.g., M00144, low dose: adj. *p* = 3.59 × 10^−6^, NES = 1.66; high dose: adj. *p* = 0.0044, NES = 1.52), mucin degradation (M10031, low dose: adj. *p* = 1.04 × 10^−5^, NES = 1.28; high dose: adj. *p* = 0.0037, NES = 1.25), vancomycin resistance (M00651, low dose: adj. *p* = 0.0430, NES = 1.27; high dose: adj. *p* = 0.0358, NES = 1.33), and L-lactate biosynthesis (M10022, low dose: adj. *p* = 0.0160, NES = −1.48; high dose: adj. *p* = 0.0008, NES = −1.78) (Figure 6).

## 4. Discussion

Numerous studies have explored natural compounds as alternative or complementary interventions for gastrointestinal discomfort, including constipation. In this context, probiotics have gained considerable attention due to their potential to support gut health [55,56,57].

Nevertheless, the efficacy of probiotics in easing constipation symptoms appears to be strain-dependent [56]. In the present study, we investigated the effects of *Bifidobacterium animalis* subsp. *lactis* CECT 8145 (BPL1^®^) on constipation symptomatology and assessed its potential laxative properties in a loperamide-induced rat model. To this end, BPL1^®^ was administered daily for three days at two doses—1.5 × 10^8^ CFU/day (low) and 3 × 10^9^ CFU/day (high)—following two subcutaneous injections of loperamide to induce constipation. Throughout the study, multiple parameters related to bowel function were evaluated, including stool frequency, stool water content, GTT, and gut microbiota composition, to comprehensively characterize the probiotic’s effects on constipation.

Loperamide-induced constipation in mice is a well-established model in the literature, as other authors have successfully modeled constipation in murine models by using loperamide [55,56,57,58]. Most studies induce constipation by administering loperamide for three days and subsequently providing the test compound, often as a single dose [10,11,58,59]. In contrast, in our study the probiotic was administered from the start of the experiment concurrently with loperamide for three days to determine whether it could attenuate or counteract the development of loperamide-induced constipation during its induction phase.

In agreement with previous studies reporting that short-term loperamide administration does not consistently alter body weight or feeding behavior in rats [10,28], our data showed no significant differences in body weight gain or cumulative food and liquid intake between control and constipated animals, irrespective of BPL1^®^ supplementation. Although some reports have described modest reductions in intake under similar experimental conditions [10,59], the absence of such effects in our study suggests that neither loperamide nor BPL1^®^ meaningfully influenced energy balance during the treatment period. Given this stability in nutritional parameters, it is reasonable to interpret the physiological changes observed in the constipated groups as primarily related to gastrointestinal function rather than alterations in appetite or energy intake.

In the present study, although no significant differences were observed in GTT between control samples, a significant decrease in stool number, fresh stool weight and humidity was observed following loperamide administration. Moreover, supplementation with both BPL1^®^ doses appears to support the increase in these parameters. Bile salt deconjugation activity, detected in *Bifidobacterium animalis* strains [60,61], could support the observed stool number increase. Metabolites derived from cholic acid degradation have been demonstrated to activate the Takeda G protein-coupled receptor 5 (TGR-5) and promote intestinal motility. Specifically, deoxycholic acid induces serotonin release via TGR-5 [62]. The BPL1^®^ genome has annotated the *bsh* gene, which is involved in the hydrolation of bile salt [25,63]. Therefore, the possible cholic acid degradation activity attributed to this gene could contribute to TGR-5 activation, which could promote serotonin-dependent intestinal motility and, ultimately, increase stool number. Other studies, such as the work of Alemi et al. (2013) [62], underscored the significance of TGR-5 activation in promoting intestinal motility in mice. Further research is needed to elucidate the broader implications of bile acid metabolism in gastrointestinal function and its potential role in easing constipation.

Similar effects to those observed in the present study were reported by other authors for *Bifidobacterium* strains. In 2021, Makizaki and collaborators reported an improvement of loperamide-induced slow transit constipation by the administration of *Bifidobacterium bifidum* G9-1 [19]. These authors pointed out that after probiotic administration in constipated animals, there was an improvement in the number of feces, fecal water content, and fecal hardness [19]. Tang T and collaborators recently reported beneficial effects on constipation for *Bifidobacterium lactis* TY-S01, including acceleration of intestinal peristalsis, maintenance of humidity of feces and prevention of the destruction of the gut barrier [56]. Nevertheless, Tang S et al. did not find increases in water content and fecal weight in loperamide-induced constipated mice treated with bifidobacteria [56].

As previously reported by other authors [10,64,65], loperamide intervention is known to alter the colon mucosa by reducing the number of goblet cells. In the present study, however, no significant differences in goblet cells count were observed among the experimental groups. To further investigate the potential mechanisms by which BPL1^®^ may exert its laxative effects on loperamide-constipated animals, the expression of different genes encoding proteins involved in intestinal fluid regulation—*mAChRM2*, *mAChRM3*, *AQP3* and *AQP8*—was analyzed. Contrary to previous reports, no significant differences were observed in the expression of these genes among the experimental groups included in this study. Moreover, considerable interindividual variability among the animals was noted, suggesting that its expression levels were not modified by either the loperamide injection or the administered intervention.

Overall, these findings indicate that the increased stool moisture observed after BPL1^®^ intervention may not be associated with changes in mucosal structure, goblet cell abundance, or gene expression, but rather with alternative physiological mechanisms. It would be interesting to explore in a future study other plausible pathways that could involve increases in luminal water content, such as the activation of the cystic fibrosis transmembrane conductance regulator (CFTR), which promotes Cl^−^ ion secretion [66]. Previous studies have shown that compounds such as flavonoids or traditional plant-based formulations can activate this channel, leading to enhanced fecal hydration in murine models [67].

Additionally, the taxonomical composition and functional pathways of the rat fecal microbiome shifted significantly depending on the condition and were altered based on the intervention. According to the PERMANOVA test results, the differences between constipated and control groups were the primary factor explaining the variability within the model at species levels. Although alpha diversity analysis between the L-Placebo and C-Placebo groups did not reveal significant differences in any of the alpha diversity measures, control animals that received the low dose of BPL1^®^ exhibited greater richness and a higher Shannon index compared to both the C-Placebo group and the constipated animals receiving the same low dose. In addition, the control group that received the high dose of BPL1^®^ showed greater richness than the C-Placebo group. Consistent with the literature, a high microbial α-diversity is generally linked to increased fiber consumption and improved metabolic health [68].

Significant taxonomical shifts were identified in the L-Placebo group, highlighting the impact of BPL1^®^ supplementation, which notably reversed some of these changes, particularly at low dose. The most striking increases were observed in the genus *Muribaculum*, the species *Muribaculum gordonibacter*, and several unclassified members of Bacteroidales (GGB24132_SGB35935 and GGB27872_SGB40306) and Bacteroidetes (GGB14001_SGB21428), among others. Both *Muribaculum* and *M. gordonibacter* belong to the *Muribaculaceae* family, which demonstrated a tendency toward greater abundance in constipated rats compared to controls. This finding aligns with previous research, which reported similar patterns in a constipation mouse model [17] and in chronically constipated humans [69]. Although the reduction at the genus and species levels was not statistically significant, a notable effect was observed at the family level. Specifically, the administration of a low dose of BPL1^®^ probiotic significantly decreased the abundance of *Muribaculaceae* in constipated animals compared to the L-Placebo group. Furthermore, the abundance of this family was significantly negatively correlated with fecal humidity.

In the loperamide-induced cohort, BPL1^®^ increased *Bifidobacterium animalis,* though only significantly at the high dose, which is likely attributable to the administration of the high dose of BPL1^®^. This increase in *B. animalis* was also found in the control comparison (C-BPL1^®^ high dose vs. C-Placebo).

Conversely, when the control animals received the high dose of BPL1^®^, a significant decrease in abundance was identified in several taxa compared to the C-Placebo group, specifically, the species *Limosilactobacilus reuteri, Lactobacillus johnsonii* and an unclassified *Lachnospiraceae* SGB41402. The intervention of certain strains of *L. reuteri* is associated with more frequent bowel movements in infants with chronic functional constipation [70]. In addition, the research of Fukushima et al., 2004 [71], suggests that *L. johnsonii* was associated with an improvement in constipation due to an increase in defecation frequency. Although a reduction in these species was observed under control conditions, no significant differences were detected in loperamide-induced constipated rats, suggesting that these species are not biomarkers of constipation in our model.

The functional analysis revealed specific KEGG functional modules enriched under the experimental conditions. In the placebo comparison, several modules were significantly enriched when comparing constipated and control animals, displaying an increase in the constipated group compared to the control group. The administration of the probiotic BPL1^®^ (at both low and high doses) seemed to revert this tendency within the loperamide induction groups compared to those receiving placebo. This positive shift was more noticeable and statistically significant with the administration of the low dose of probiotic BPL1^®^.

Some of the modules affected were related to vancomycin resistance (M00652) and mucin degradation (M10031). These modules were much lower when the low dose of BPL1^®^ was administered to constipated rats compared to the placebo. These findings align with the literature; Gao et al. [72] reported that constipation is associated with reduced mucus production, characterized by a significant drop in mucin and a thinner inner mucus layer. The compromised mucus layer protects the gut barrier and lubricates it by keeping the mucosal surface moist. A thinner layer could make the gut more susceptible to inflammation. Separately, vancomycin-resistance genes (M00652) are found in various pathogenic bacteria and are clinically important due to their role in glycopeptide antibiotic resistance [73]. Although these functional modules in the L-BPL1^®^ high-dose group did not reach statistical significance when compared to the L-placebo group, they followed the same tendency observed in the L-BPL1^®^ low-dose comparison.

In addition, several other modules also followed this tendency in that comparison, including isoleucine biosynthesis (M00019 and M00570) and CMP-KDO biosynthesis (M00063), among others. These results were supported by the existing literature suggesting that intervention with this probiotic may also improve constipation by functional modulation of the microbiota. Increased enrichment in the L-isoleucine pathway [74] and metabolites [75] observed in functional constipation subjects compared to controls may be because of malabsorption due to epithelial inflammation [75]. No literature links the CMP-KDO biosynthesis (M00063) module with constipation. However, CMP-KDO biosynthesis is necessary for lipopolysaccharide (LPS) synthesis in Gram-negative bacteria (e.g., *Escherichia coli* [76]). LPS can stimulate the production of pro-inflammatory cytokines such as tumor necrosis factor (TNF)-α and interleukin (IL)-6) by macrophages via the TLR4/nuclear factor kappa B (TNF-κB) pathway, thereby inducing systemic inflammation [77]. In this review, the authors explore how gut microbiota (including LPS from Gram-negative bacteria) could influence colonic motility. However, the connection between constipation and inflammation is something that researchers continue to investigate in order to gain more insight into the matter. Moreover, administration of the low dose of BPL1^®^ to the constipated model resulted in increased abundance of the biosynthesis of L-lactate (M10022) and Pyridoxal-P (M00916) compared to the placebo. Lactic acid bacteria produce lactate and acetate, which promote gut secretion, enhancing peristalsis and helping to soften the stool [78], again related to the promotion of colonic serotonin production by the interaction with the enterochromaffin cells [72]. Vitamin B6, also known as pyridoxine and pyridoxal, can be incorporated through diet and may help to ameliorate constipation symptoms by improving gut motility and softening stool [79]. In addition, Vitellio et al. [80] found that taking probiotics in combination with vitamin B6 could be helpful to reduce bloating and improve constipation.

This pilot study provides preliminary evidence that the BPL1^®^ probiotic may alleviate loperamide-induced constipation through different mechanisms. While these findings are promising, the exploratory nature of the study—particularly its limited sample size and short intervention period—requires thoughtful interpretation. To substantiate and expand upon these results, future research should involve larger, more representative cohorts and longer intervention durations, ideally complemented by comprehensive physiological assessments to strengthen the robustness of the conclusions.

## 5. Conclusions

This exploratory study suggests that the intervention with *Bifidobacterium animalis* subsp. *lactis* CECT 8145 BPL1^®^ probiotic was associated with an improvement in loperamide-induced constipation in a murine model, potentially related to multiple mechanisms. We documented improvements in stool excretion and consistency, and fecal humidity, recovering the effect induced by loperamide. These benefits were tied to changes in gut microbiota composition, characterized by a decrease in *Muribaculaceae* and *Muribaculum gordoncarteri* abundance and in metabolic pathways, revealing a reduction in functional modules associated with mucin degradation, antibiotic resistance to vancomycin and isoleucine biosynthesis, as well as an enrichment of L-lactate and Pyridoxal-P biosynthesis, which may contribute to constipation relief. Despite the limitations regarding sample size and study duration, these preliminary findings provide a promising basis for future preclinical and clinical studies to evaluate the efficacy of BPL1^®^ in constipation.

## Figures and Tables

**Table 1:** Nucleotide sequences of primers used for PCR amplification in colon samples.

Gene	Forward Primer (5′ to 3′)	Reverse Primer (3′ to 5′)
*mAChRM2*	CCAAAAGGGTGATGTGTGCA	AGCCAAGATTGTCCTGGTCA
*mAChRM3*	AGCAGGAGTCAGAACCCTTC	GCCAGAAGAATGAGAGCAGC
*AQP3*	ACCCAGGAGTGCGTTTCTAA	GGACTTTAGCCCCTCCCAAT
*AQP8*	AGGGGAAGGAGACCAACATG	CACAGCAGGGTTGAAGTGTC
*PPIA*	CCAAACACAAATGGTTCCCAGT	ATTCCTGGACCCAAAACGCT

**Table 2:** Measurement of body weight gain and feeding behavior in non-constipated (C) and constipated (L) rats.

	Feeding Behavior		
Groups	Body Weight Gain (g)	Cumulative Food Intake (g)	Cumulative Liquid Intake (mL)
C-Placebo	14.43 ± 0.94	54.85 ± 1.08	207.33 ± 16.34
C-BPL1™ Low dose	12.15 ± 1.17	52.66 ± 1.59	211.86 ± 20.11
C-BPL1™ High dose	12.03 ± 1.85	52.35 ± 1.37	218.43 ± 21.41
L-Placebo	11.48 ± 1.02	50.35 ± 1.75	201.28 ± 14.55
L-BPL1™ Low dose	11.79 ± 1.25	53.21 ± 1.45	243.38 ± 22.01
L-BPL1™ High dose	11.07 ± 1.84	49.63 ± 2.12	207.65 ± 22.51

All values are presented as estimated marginal means ± SEM. C-Placebo: placebo, control; C-BPL1^®^ Low dose: BPL1^®^ low dose, control; C-BPL1^®^ High dose: BPL1^®^ high dose, control; L-Placebo: placebo, loperamide; L-BPL1^®^ Low dose: BPL1^®^ low dose, loperamide; L-BPL1^®^ High dose: BPL1^®^ high dose, loperamide.

**Table 3:** Water content in 8 h record in non-constipated (C) and constipated (L) rats.

Water Content (%)	0–2 h	2–4 h	4–6 h	6–8 h
C-Placebo	59.27 ± 6.03	53.49 ± 1.89	48.86 ± 8.09	47.11 ± 1.74
C-BPL1^®^ Low dose	57.95 ± 1.35	54.90 ± 2.16	57.44 ± 3.07	53.15 ± 0.34
C-BPL1^®^ High dose	56.67 ± 1.78	54.06 ± 2.53	-	68.84 ± 10.81
L-Placebo	46.51 ± 6.34	54.58 ± 3.34	48.13 ± 4.57	-
L-BPL1^®^ Low dose	58.32 ± 2.90	-	48.19 ± 1.34	-
L-BPL1^®^ High dose	56.94 ± 2.65	53.67 ± 3.02	49.11 ± 2.08	-

All values are presented as estimated marginal means ± SEM. (-) Data not available. C-Placebo: placebo, control; C-BPL1^®^ Low dose: BPL1^®^ low dose, control; C-BPL1^®^ High dose: BPL1^®^ high dose, control; L-Placebo: placebo, loperamide; L-BPL1^®^ Low dose: BPL1^®^ low dose, loperamide; L-BPL1^®^ High dose: BPL1^®^ high dose, loperamide.

**Table 4:** Gastrointestinal transit time in control and loperamide rats.

Groups	GTT (Hours)
C-Placebo	13.30 ± 0.47
C-BPL1^®^ Low dose	12.60 ± 0.54
C-BPL1^®^ High dose	12.60 ± 0.56
L-Placebo	14.30 ± 0.40
L-BPL1^®^ Low dose	13.20 ± 0.44
L-BPL1^®^ High dose	14.22 ± 0.43

All values are presented as estimated marginal means ± SEM. Results of *t*-test are shown. C-Placebo: placebo, control; C-BPL1^®^ Low dose: BPL1^®^ low dose, control; C-BPL1^®^ High dose: BPL1^®^ high dose, control; L-Placebo: placebo, loperamide; L-BPL1^®^ Low dose: BPL1^®^ low dose, loperamide; L-BPL1^®^ High dose: BPL1^®^ high dose, loperamide.

**Table 5:** Histological measurements in transversal colon from control and loperamide rats. GALT, gut-associated lymphoid tissue.

Groups	Goblet Cells (%)	GALT	Germinal Centers
C-Placebo	64.10 ± 2.83	1.10 ± 0.31	0.60 ± 0.16
C-BPL1^®^ Low dose	60.20 ± 2.78	0.90 ± 0.28	0.50 ± 0.17
C-BPL1^®^ High dose	60.60 ± 1.34	0.70 ± 0.26	0.40 ± 0.16
L-Placebo	61.80 ± 1.31	0.80 ± 0.29	0.50 ± 0.17
L-BPL1^®^ Low dose	67.70 ± 3.73	1.10 ± 0.31	0.60 ± 0.16
L-BPL1^®^™ High dose	67.60 ± 2.45	0.80 ± 0.33	0.40 ± 0.16

All values are presented as estimated marginal means ± SEM. Results of *t*-test are shown. C-Placebo: placebo, control; C-BPL1^®^ Low dose: BPL1^®^ low dose, control; C-BPL1^®^ High dose: BPL1^®^ high dose, control; L-Placebo: placebo, loperamide; L-BPL1^®^ Low dose: BPL1^®^ low dose, loperamide; L BPL1^®^ High dose: BPL1^®^ high dose, loperamide.

## Data Availability

The data that support the findings of this study are available from the corresponding author upon reasonable request due to restrictions associated with proprietary material.

## Supplementary Materials

The following supporting information can be downloaded at: https://www.mdpi.com/article/10.3390/nu18081237/s1, Figure S1: Bar plot at species level with the relative abundance (%) of each species in each sample; Figure S2: (A) Alpha diversity violin plots at species level with richness, Shannon and Simpson indexes according to the groups. (B) PCoA graph of samples at species levels, colored according to the groups compared; Figure S3: Normalized count distribution of the family *Muribaculaceae*: (a) using boxplots with logarithmic counts and separated according to the condition and intervention variables and (b) negative correlation between the normalized counts of *Muribaculaceae* and humidity (%). (*): *p*-value < 0.05; Figure S4: Complex heatmap with the genus differentially abundant according to the log2FC of the group compared. Red color indicates that the genus is over-represented in the first group of the comparison, while blue color indicates it is over-represented in the second group of the comparison. The barplot shows the mean normalized abundance (baseMean) of each taxon. (*): adj *p*-value < 0.05.

## Abbreviations

The following abbreviations are used in this manuscript:

ACh	Acetylcholine
AQP	Aquaporin
bw	Body weight
CAZy	Carbohydrate active enzymes
cDNA	Complementary DNA
CFU	Colony-forming unit
FDR	False discovery rate
GALT	Gut-associated lymphoid tissue
GI	Gastrointestinal
GSEA	Gene set enrichment analysis
GTT	Gastrointestinal transit time
H&E	Hematoxylin and eosin
mAChR(s)	Muscarinic acetylcholine receptor(s)
NES	Normalized enrichment score
ORF	Open reading frames
PCoA	Principal coordinates analysis
PPIA	Peptidylprolyl isomerase A
SCFAs	Short-chain fatty acids
SD	Sprague–Dawley
TGR-5	Takeda G protein-coupled receptor 5
