# Supplementary material for: Bifidobacterium animalis subsp. lactis CECT 8145 BPL1® Laxative Effects in Loperamide-Induced Constipated SD Rats
Source: Nutrients. 2026 Apr 14;18(8):1237. doi: 10.3390/nu18081237 (PMC13118976; doi:10.3390/nu18081237)
Supplement: Supplementary file 1 [file nutrients-18-01237-s001.zip › nutrients-4195769-supplementary.pdf]

Supplementary materials

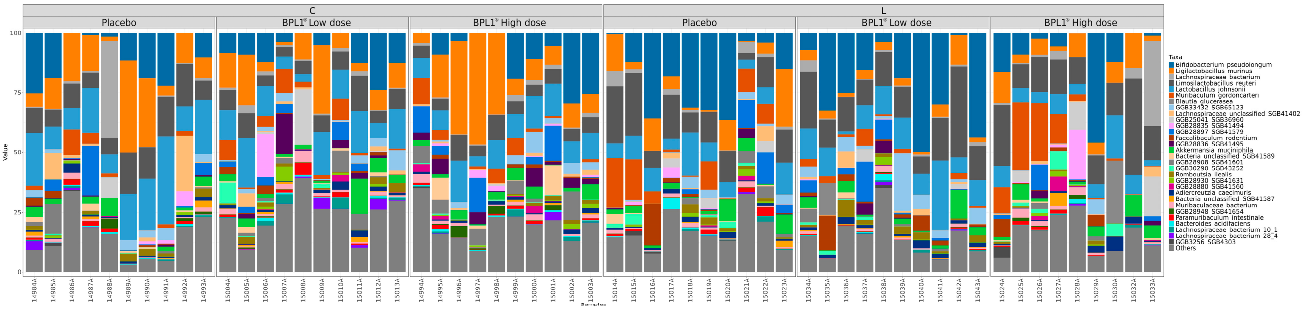

Supplementary Figure S1. Bar plot at species level with the relative abundance (%) of each species in each sample.

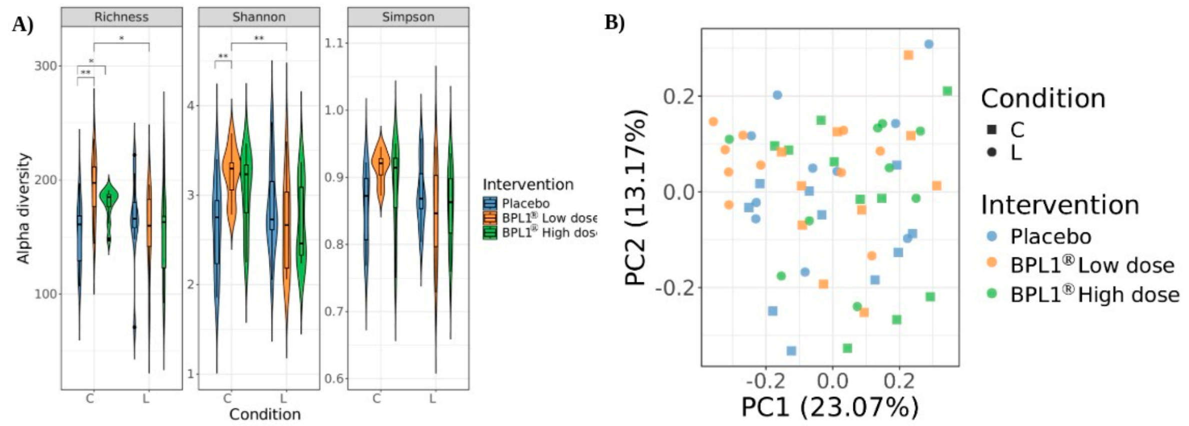

Supplementary Figure S2. Alpha diversity violinplots at species level with Richness, Shannon and Simpson indexes according to the groups (**A**). PCoA graph of samples at species levels, coloured according to the groups compared (**B**).

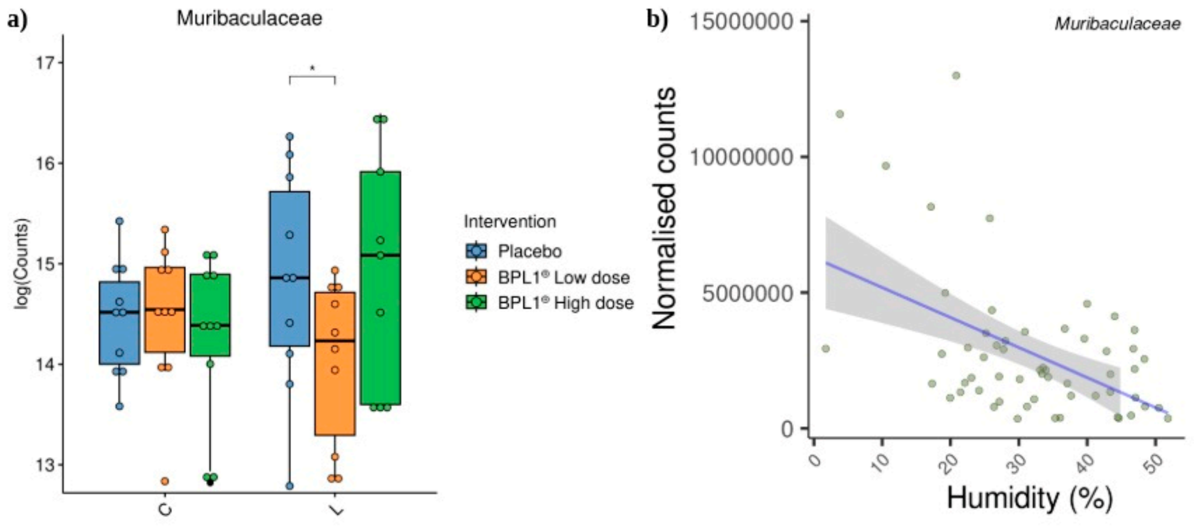

Supplementary Figure S3. Normalised counts distribution of the family *Muribaculaceae*: a) using

boxplots with logarithmic counts and separated according to the Condition and Intervention variables and b) negative correlation between the normalised counts of *Muribaculaceae* and humidity (%). (*): *p*-value <0.05.

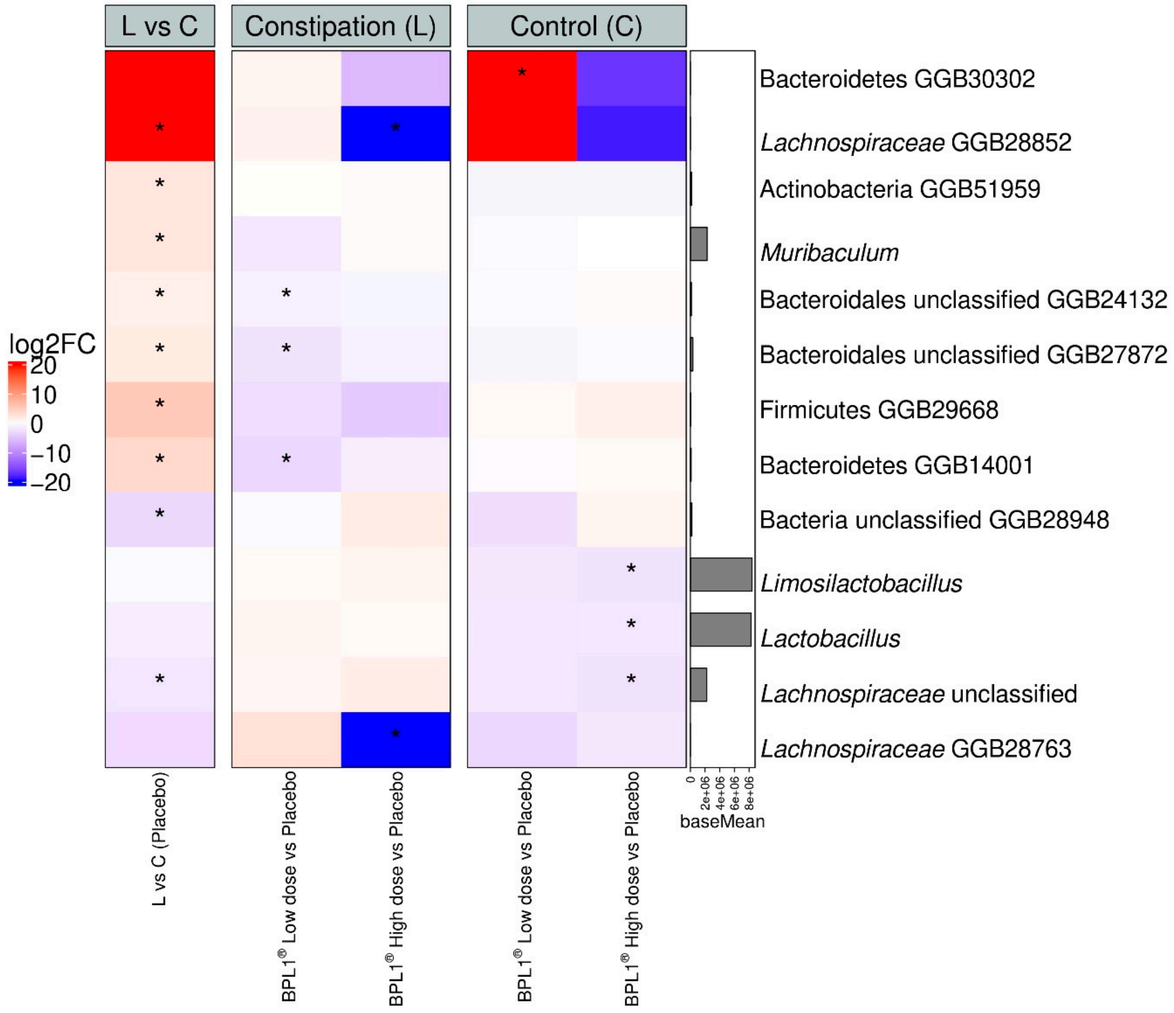

Supplementary Figure S4. Complex Heatmap with the genus differentially abundant according to the log2FC of the group compared. Red colour indicates that the genus is over-represented in the first group of the comparison, while blue colour indicates it is over-represented in the second group of the comparison. The barplot shows the mean normalized abundance (baseMean) of each taxon. (*): adj *p*-value <0.05.
